# Gastric and pulmonary lymphoma presenting as a solitary pulmonary nodule

**DOI:** 10.2349/biij.3.4.e51

**Published:** 2007-10-01

**Authors:** EL Thomas, NP Lenzo, R Troedson

**Affiliations:** 1 Department of Nuclear Medicine, Division of Medical Imaging, Royal Perth Hospital, Perth, Australia; 2 School of Medicine, Notre Dame University Fremantle Campus, Fremantle, Australia; 3 Department of Nuclear Medicine, Princess Margaret Hospital for Children, Subiaco, Australia; 4 WA PET/Cyclotron Service, Nedlands, Australia

**Keywords:** FDG-PET, Non-Hodgkin’s Lymphoma, solitary pulmonary nodule

## Abstract

The common presentations of lymphoma are widespread lymphadenopathy or development of constitutional symptoms. This paper presents a case of a patient who presented with a solitary mass detected on chest X-ray and underwent FDG-PET for further evaluation of this mass. FDG-PET is a commonly utilised technique to assess solitary nodules as it not only allows characterisation of the lesion but can also detect nodal and extra-thoracic disease with greater accuracy than the standard CT. In this case, FDG-PET demonstrated abnormal activity in the lung nodule and at the gastro-oesophageal junction. Biopsies confirmed Non-Hodgkin’s Lymphoma at both sites. The value of FDG-PET in this case was the determination of previous unsuspected disease in an unusual presentation of lymphoma and as a useful tool for monitoring the therapeutic effect post chemotherapy.

## LEARNING POINTS

FDG-PET:

- is of clinical benefit in the assessment of the solitary pulmonary nodule;
- can often detect occult metastatic disease; and
- is a sensitive modality for monitoring therapeutic efficacy in lymphoma.

## CASE

A 71-year-old lady had an incidental finding of an opacity in her left lower lobe on a chest radiograph. Thoracic CT confirmed the presence of a 1.0 cm solitary pulmonary nodule but did not identify any lymphadenopathy or evidence of distant metastatic disease.

**Figure 1:**
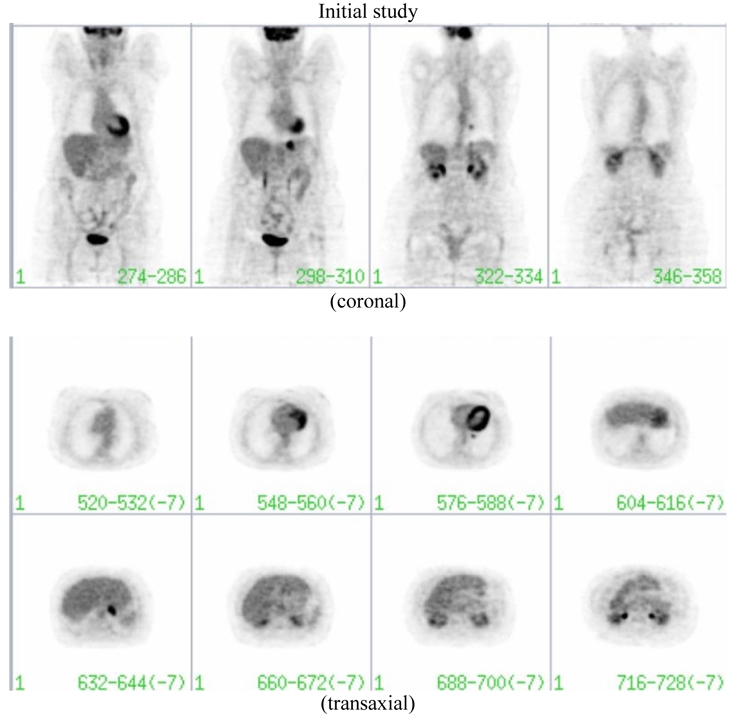
The PET study (Philips Allegro system) reveals a small retrocardiac focus of increased FDG uptake within the left lower lobe nodule (thin arrow) suggestive of a malignant process. In addition, the study demonstrates abnormal increased activity at the gastro-oesophageal junction (thick arrow).

The patient was referred for an F18 FDG-PET study (Figure 1) for investigation of the pulmonary nodule [1]. The PET study confirmed a small retrocardiac focus of increased FDG uptake within the left lower lobe nodule (thin arrow) suggestive of a malignant process. In addition, the study demonstrated abnormal increased activity at the gastro-oesophageal junction (thick arrow). It was proposed that metastasis at this site from a primary lung malignancy would be unusual and that two malignancies (i.e. lung and gastric) were possible [2]. Lung metastasis from a gastric neoplasm was also possible, however, isolated pulmonary metastasis from gastric cancer is rare. Thus, further investigation was recommended.

The patient underwent upper gastrointestinal endoscopy. Biopsy of the gastric wall during endoscopy revealed non-Hodgkin’s lymphoma (NHL). The patient proceeded to thoracotomy for investigation of her lung lesion to definitively ascertain the nature of this lesion. Pathology again confirmed lung NHL. Therefore, the patient was treated with systemic chemotherapy for lymphoma.

**Figure 2:**
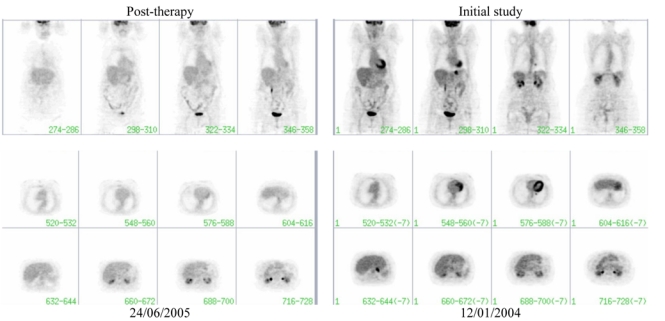
A follow-up FDG-PET study post therapy (left) shows a complete metabolic response to treatment both in the stomach and the left lung when compared to the initial study (right).

A follow-up post therapy FDG-PET study (Figure 2, right) showed a complete metabolic response to treatment both in the stomach and the left lung when compared to the initial study (Figure 2, left).

## DISCUSSION

Non-Hodgkin’s lymphoma is a known but relatively rare cause of solitary pulmonary nodules [3]. Gastrointestinal lymphoma can be FDG-avid but can be difficult to differentiate from normal stomach activity [4]. Likewise, it can be difficult to appreciate with anatomical imaging methods such as CT. In this case, however, the gastric uptake of FDG was focal and somewhat more intense than is usually seen at the gastro-oesophageal junction. This led to the suspicion of a pathological process at this site, which was subsequently confirmed at biopsy. Lymphoma is usually characterised by nodal involvement on both CT and FDG-PET. Extranodal disease is less common. The unusualness of this presentation necessitated tissue confirmation both from the stomach and the lung nodule.

FDG-PET is used throughout the world in the assessment of solitary pulmonary nodule. It has been shown to have high sensitivity (>95%) and high specificity (>75%) in determining whether the nodule is benign or malignant based on high uptake in the lesion [5]. In this case, FDG-PET confirmed a likely malignant process with prominent activity seen in the small lung nodule.

FDG-PET in this case also showed a total metabolic response to treatment following appropriate chemotherapy. In the authors' institution, post-therapy assessment in NHL is one of the most common indications for FDG-PET. The findings may be of prognostic significance as total metabolic response has been associated with improved long term outlook in NHL [6]. FDG-PET has also been shown to be more accurate in assessing response to therapy in NHL compared with CT [7]. The whole body nature of PET imaging coupled with the sensitivity related to its assessment of metabolic function rather than anatomic detail likely contribute to its enhanced accuracy compared to CT.

This case thus illustrates the usefulness of FDG-PETas teaching points:

- in the assessment of the solitary pulmonary nodule
- in the detection of previously occult disease and
- as a sensitive modality for monitoring therapeutic efficacy in lymphoma.
